# Pathogenicity of the First Buffalo-origin Senecavirus A in Conventional Piglets and Buffaloes

**DOI:** 10.1155/tbed/6222217

**Published:** 2025-09-02

**Authors:** Xia Zhou, Qi Zhai, Jiawei Niu, Gen Li, Tianbao Chen, Yan Li, Huahua Kang, Chunling Li, Hongchao Gou, Pinpin Chu, Kunli Zhang, Zhiyong Jiang, Zhibiao Bian, Ming Liao, Shao-Lun Zhai

**Affiliations:** ^1^Institute of Animal Health, Guangdong Academy of Agricultural Sciences/Guangdong Provincial Key Laboratory of Livestock Disease Prevention/Scientific Observation and Experiment Station of Veterinary Drugs and Diagnostic Techniques of Guangdong Province, Guangzhou, Guangdong, China; ^2^College of Veterinary Medicine, South China Agriculture University, Guangzhou, Guangdong, China; ^3^National and Regional Joint Engineering Laboratory for Medicament of Zoonosis Prevention and Control, Guangdong Provincial Key Laboratory of Zoonosis Prevention and Control, Guangzhou, Guangdong, China

**Keywords:** buffalo, buffalo-origin, experimental infection, pathogenicity, piglet, Senecavirus A

## Abstract

As the only member of the genus *Senecavirus* within the family *Picornaviridae*, Senecavirus A (SVA) has posed an enormous challenge for the pig industry worldwide. In our previous study, a SVA strain was isolated from a buffalo with mouth ulcers. To systematically assess its pathogenicity, this study compared the outcome of piglets and buffaloes artificially infected by the different viral dose of the buffalo-origin SVA strain (SVA/GD/China/2018). These results indicated that vesicular diseases can occur in infected piglets and buffaloes. Severe clinical symptoms were observed in the piglets and buffaloes with the inoculation of 10^5.0^ 50% tissue culture infective dose (TCID_50_/mL). The SVA antigen expression was also detected in the lung tissue, chin blister lesion tissue, nasolabial tissue of the piglets, and the upper lip blister tissue of the buffaloes. This study demonstrated that the buffalo-origin SVA strain was pathogenic to piglets and buffaloes, revealing the possibility of cross-species transmission of SVA between pigs and buffaloes. In the future, it is necessary to strengthen the surveillance of SVA in cattle herds.

## 1. Introduction

In 2002, Senecavirus A (SVA), or called Seneca Valley virus (SVV), an emerging RNA virus belonging to the *Senecavirus* genus, was found in a cell culture contaminant, and it was suspected to be introduced via porcine trypsin or fetal bovine serum (FBS) which are commonly used in cell culture [1]. Since then, SVA has been confirmed to be a causative agent of vesicular disease in swine, similar to foot-and-mouth disease virus (FMDV). Since 2015, SVA has spread to Brazil, the United States, China, Colombia, Thailand, Vietnam, and Chile [2–9]. Most cases occurred in Brazil, the United States, and China [3, 10, 11].

In addition to pigs, SVA can be detected in mice, houseflies, and *Culicoides* [10, 12, 13]. Interestingly, viable SVA was recovered from mouse feces and small intestines, suggesting that these pests play an important role in the transmission of SVA [10]. Apart from pigs, the other infected natural hosts have not to be elucidated. In 2018, we isolated a SVA strain, SVA/GD/China/2018, from a buffalo with mouth ulcers in the Guangdong Province of China, which revealed that buffalo could be another host of SVA [14]. To systematically assess its pathogenicity, in this study, experimental infection of conventional piglets and buffaloes with the buffalo-origin SVA was performed.

## 2. Materials and Methods

### 2.1. Viral Strain

SVA strain, SVA/GD/China/2018 (GenBank accession number: MN615881) had been isolated from China, in 2018 and stored at the Institute of Animal Health, Guangdong Academy of Agricultural Sciences [14]. Viruses were generated viral stocks at titers of 10^8.0^, 10^5.0^ and 10^3.0^ 50% tissue culture infective doses (TCID_50_/mL). Simply, BHK-21 cells cultured in Dulbecco′s modified Eagle′s medium (DMEM) supplemented with 2% FBS (Thermo Fisher Scientific) were grown at 37°C under 5% CO_2_ and employed to isolate SVA. The SVA were titrated with Reed-Muench method in 96 well cell plates.

### 2.2. Animals and Experimental Design

A total of 11 40-day-old piglets were purchased from Yangshan Agricultural Company in Guangdong Province. All the conventional piglets were tested negative for FMDV, swine vesicular disease virus (SVDV), porcine reproductive and respiratory syndrome virus (PRRSV), pseudorabies virus (PRV), classical swine fever virus (CSFV), and SVA. Eleven piglets were randomly divided into four groups and inoculated with different TCID_50_, including group A (10^8.0^ TCID_50_/mL, *n* = 3), group B (10^5.0^ TCID_50_/mL, *n* = 3), group C (10^3.0^ TCID_50_/mL, *n* = 3), and control group D (DMEM, *n* = 2). The piglets in groups A–C were inoculated intranasally (1.5 mL per nostril), orally (3 mL), and intramuscularly (3 mL) with the SVA/GD/China/2018 strain, respectively. Moreover, the piglets in group D were inoculated with DMEM (pH = 7.4, Gibco, USA) via the same routes in the containment animal facility. The piglets in groups A–C were housed in three different containers separately, and the piglets in group D were placed in another room. After infection, clinical signs of illness daily, including mental status or appetite, breathing problems, the occurrence and development of lesions, were observed and recorded for all the piglets. Based on the other references, clinical scoring (CS) was conducted according to Table 1 [15, 16].

In addition, the rectal temperature and body weight were recorded at 2, 4, 6, 8, 10, and 12 days post inoculation (dpi). Oral, nasal, and fecal swabs were collected every 2 dpi and the SVA VP1 gene copies were detected via real time quantitative reverse transcription polymerase chain reaction (qRT-PCR) after extraction of viral RNA. Furthermore, the swab samples were used to isolate SVA from the swine testicular (ST) cell line. All surviving piglets were euthanized and necropsied at 14 dpi.

Nine buffaloes aged from 110 to 180 days were purchased from a buffalo farm in Sihui city, Guangdong province. All buffaloes were tested and found to be free of FMDV, vesicular stomatitis virus (VSV), bovine viral diarrhea virus (BVDV), bluetongue virus (BTV), brucella, and SVA. Nine buffaloes were randomly divided into three groups. The buffaloes in groups A–C were inoculated with SVA/GD/China/2018 strain containing 10^8.0^ TCID_50_/mL, 10^5.0^ TCID_50_/mL, and DMEM, respectively. All buffaloes from groups A and B were inoculated intranasally (2.5 mL per nostril), orally (5 mL), and intramuscularly (5 mL) with different virus stocks, and the buffaloes in group C were inoculated with DMEM via the same routes and at the same dose in the containment animal facility. During the experiment, clinical signs of illness, including mental status, respiratory signs, mouth, nose, oral mucosa, and coronary vascular zone damage, were observed and recorded daily. Each blister was regarded as a scoring object. The average scores of lesion were calculated daily for each group following the three classification points in Table 1 [17]. Oral, nasal, and fecal swab samples were collected from the buffaloes daily after 2 dpi for detecting SVA genome copies via qRT-PCR. One seriously infected buffalo was euthanized and necropsied at 7 dpi, and the heart, liver, spleen, lung, kidney, rumen, reticulum, valved stomach, abomasum, duodenum, jejunum, ileum, blind gut, colon, and rectum tissues were collected for qRT-PCR analysis and immunohistochemistry (IHC). SVA was isolated again from the oral and nasal swab samples using ST cell lines.

All of the experiments were performed at the Laboratory Animal Care of the Institute of Animal Health, Guangdong Academy of Agricultural Sciences. The experiments were approved and conducted per the relevant guidelines with strict biosecurity measures and hygiene procedures.

### 2.3. RNA Extraction and One-step Real-time Quantitative PCR

The collected samples were diluted with 1 mL of phosphate-buffered saline (PBS, pH = 7.4, Gibco, USA) and vortexed by centrifugation, and 200 µL of the cleared sample was used for detecting SVA VP1 gene copies. Total RNA was extracted from the samples with an AxyPrep Body Fluid Viral DNA/RNA Miniprep Kit (Union City, CA, USA) following the manufacturer's protocol. SVA gene copies were generated using qRT-PCR targeting parts of the VP1 gene. The qRT-PCR forward and reverse primers were 5′–GAGGCAGGTAACACTGACAC–3′ and 5′–GCGTCCTTCTCCAGT ACCTT–3′, respectively. The TaqMan probe was synthesized as 5′–CTCTGGTGAAC TGGCGGCTCCTGGC–3′ with 5′ modified by FAM and 3′ modified by BHQ1. All the designed primers and TaqMan probe were synthesized by Sangon Biotech (Shanghai, China). Samples with a cycle threshold (Ct) value of < 35 were considered as positive. All qRT-PCR analyses for SVA were performed at the Institute of Animal Health, Guangdong Academy of Agricultural Sciences.

### 2.4. IHC

The organ samples were fixed with a 10% buffered formalin solution, and routine paraffin sections were prepared [17]. Sections were dewaxed with xylene I, II, and III (Fuyu Chemical, Tianjin, China) for 15 min orderly and then hydrated with absolute ethanol (Fuyu Chemical, Tianjin, China) for 5 min, 85% alcohol (Fuyu Chemical, Tianjin, China) for 5 min, and 75% alcohol for 5 min, orderly. Then, the sections were rinsed in distilled water. Sections were placed in a repair box filled with citric acid antigen retrieval buffer (pH = 6.0) for antigen retrieval in a microwave oven. After natural cooling, the sections were placed in PBS and shaken on a decolorization shaker three times for 5 min each. Then, the sections were placed in 3% hydrogen peroxide, incubated at room temperature in darkness for 25 min, placed in PBS (pH = 7.4), and shaken on a decolorizing shaper three times for 5 min each to block endogenous peroxidase activity. Then, 3% bovine serum albumin (BSA, Sangon Biotech, Shanghai, China) was added to the circle to cover the tissues evenly, and the tissues were sealed for 30 min at room temperature. The sealing solution was gently removed. The SVA VP3 primary antibody was added to the sections at a 1:300 dilution. The sections were placed flat in a wet box and incubated overnight at 4°C. After the sections were washed 5 min each time by shaking on a decolorizing shaker for three times, the sections were incubated with the secondary antibody HRP-Goat anti-Mouse IgG (Servicebio, Wuhan, China) for 50 min at room temperature. Next, the newly prepared DAB color-developing solution (Servicebio, Wuhan, China) was used for chromogenic reactions. The nuclei were counterstained with hematoxylin stain solution (Servicebio, Wuhan, China) for approximately 3 min. The sections were dehydrated with alcohol and mounted with neutral gum. Finally, the sections were visualized under a microscope. The positive cells were brownish-yellow [18].

### 2.5. Virus Isolation

ST cell line was used for SVA isolation from the oral and nasal swabs of piglets and buffaloes infected with SVA at 6 dpi. The swabs were fully mixed and infiltrated into a PBS without Mg^2+^ and Ca^2+^ (pH = 7.0). The filtrates were incubated on ST monolayer cell line after filtering with 0.22 μM filters for 1 h. Then, the mixture was removed, and DMEM supplemented with 2% FBS was used instead of incubating until a cytopathic effect (CPE) occurred. Each sample was passaged blindly three times. At the same time, the mock treatment was also administered. The ST cell lines and the SVA isolates, SVA/GD/China/2018, were preserved in our laboratory.

### 2.6. Immunofluorescence Assay (IFA)

For the IFA, ST cells infected with SVA were washed three times with PBS and fixed in 4% paraformaldehyde solution (PFA, Servicebio, Wuhan, China) for 10 min at room temperature, and then ST cells were permeabilized with PBS/0.1% Triton X-114 (Sigma–Aldrich, St. Louis, MO, USA) and blocked in 5% BSA in PBS. Next, ST cells were incubated with the primary antibodies specific for SVA VP3 protein (1:100 dilution in PBS/0.1% Triton X-114). Then, ST cells were incubated overnight at 4°C. After washing three times with PBST dilution, the cells were incubated light-avoidedly at 37°C for 1 h with the secondary antibodies (goat anti-mouse IgG labeled with FITC, 1:500, Proteintech, China). After the secondary antibodies was washed three times with PBST dilution, the nucleus of ST cells were stained and blocked with an anti-fluorescence quenching blocking agent with DAPI (Solarbio, China). Immediately, Life Tech EVOS FL Auto Fluorescence Microscope System (Thermo Fisher Scientific, USA) was used for imaging under 40× magnification.

### 2.7. Statistical Analysis

Statistical analysis and data plotting were performed using GraphPad Prism (version 6.0) software (GraphPad Software, Inc., La Jolla, CA). The differences in the data were determined using *t*-tests, Student's *t*-tests, Welch's *t*-tests, or Mann–Whitney *U*-tests; one-way ANOVA for multiple comparisons; and Tukey's or Sidak's multiple-comparison-test, as applicable and according to the test requirements. A *p*-value < 0.05 was considered to indicate statistical significance and was marked with asterisks and “ns”: *⁣*^*∗*^*p* < 0.05, *⁣*^*∗∗*^*p* < 0.01, and *⁣*^*∗∗∗∗*^*p* < 0.0001.

## 3. Results

### 3.1. Clinical Manifestations and Symptom Scores

A total of 11 piglets and nine buffaloes were included in this study. All of them met the selection criteria. After virus infection, the piglets in the three infection groups and the buffaloes in the two infection groups exhibited varying clinical symptoms.

**Figure 1:**
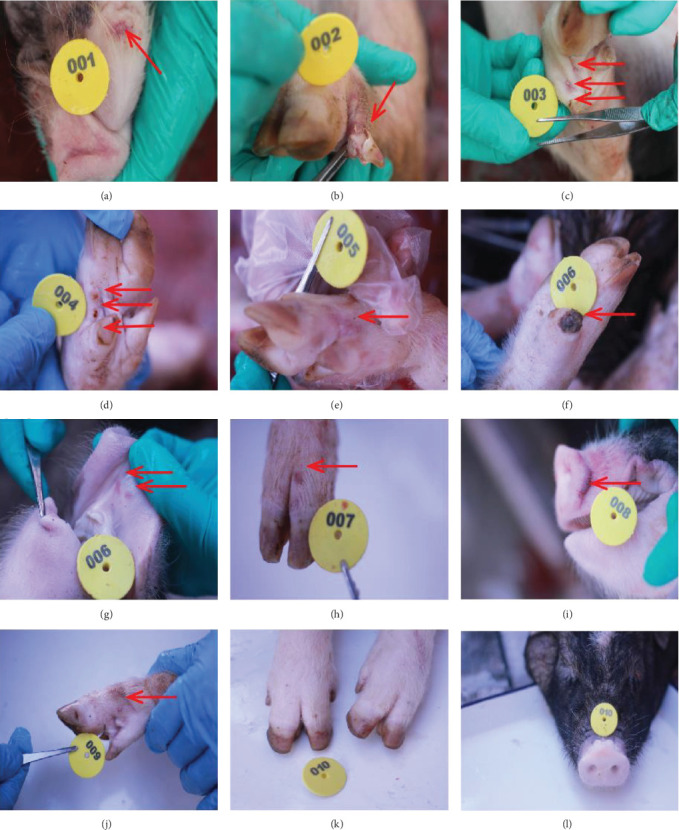
Vesicular lesions were observed on piglets infected with 10^8.0^ (A–C), 10^5.0^ (D–G), and 10^3.0^ (H–J) TCID_50_/mL SVA. Vesicular lesions were observed on the lower lip (A) and hoof nails (B–H) and snout (I). Fell off toenails (B) and putrid toenails (F) happened separately on the number 002 piglet from group A (10^8.0^ TCID_50_/mL) and the number 006 piglet from group B (10^5.0^ TCID_50_/mL). More blisters, rotted, and shedding hooves were happened on the experimental piglets from groups A (A–C) and B (D–G), which were more than that of the experimental piglets from group C (H–J). Piglets inoculated with the DMEM did not present any vesicular lesion on the hoof nails (K) and snout (L) throughout the experiment.

After experimental infection, for the piglets (number 004–006) in group B, the vesicles first developed with multiple blisters at the toenails and lips as shown in Figure 1D–G. Seriously, one toenail of the pig number 006 even rotted off. Compared with those piglets in group B, the clinical symptoms of piglets (number 001–003) in group A, included fewer blisters on hoof nails. Additionally, the hoof nail of one piglet (number 002) also fell off, as shown in Figure 1. For the piglets in group C (number 007–009), only a few sporadic blisters appeared on the nose of one piglet (number 008), as shown in Figure 1I and on the hoof nails of two piglets (number 007 and 009), as shown in Figure 1H, J. The piglets of control group (number 010 and 011) had no blisters (Figure 1K, L).

**Figure 2:**
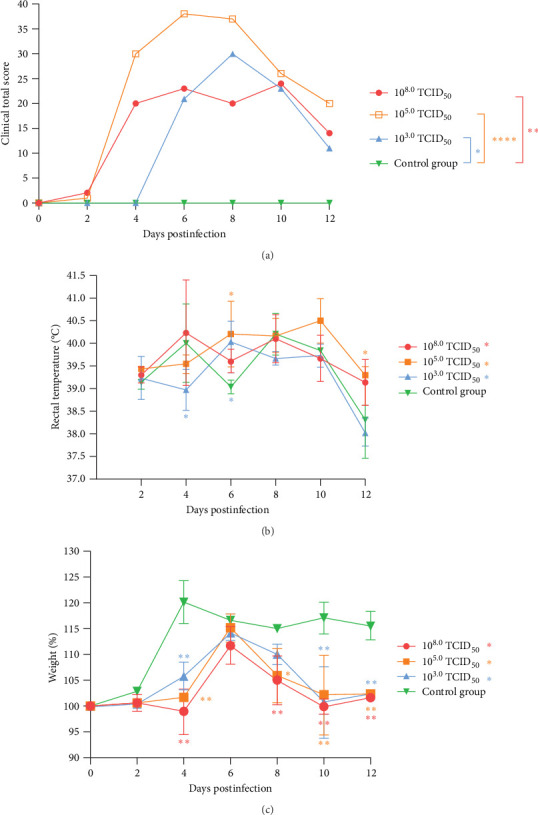
Evaluation of clinical data. The score of piglets from group B (10^5.0^ TCID_50_/mL) was more than 30 between 4 and 9 dpi, and others were below 30 (A). The rectal temperature of piglets from group B (10^5.0^ TCID_50_/mL) was higher than that of groups A (10^8.0^ TCID_50_/mL) and C (10^3.0^ TCID_50_/mL) (B). The weight growth rates of piglets in groups A–C were even lower than those in the control group (C). Significant differences are marked with asterisks: *⁣*^*∗∗*^*p* < 0.01; *⁣*^*∗*^*p* < 0.05.

Moreover, clinical symptom scores were recorded at 2, 4, 6, 8 and 10 dpi, respectively. The score results are shown in Figure 2A. The clinical symptoms firstly appeared at 2 dpi in piglets from groups A and B, and the scores reached the highest values at 6 dpi. For the piglets in group C, clinical symptoms appeared only at 4 dpi, and the score reached the highest value at 8 dpi and then gradually decreased until the end of the experiment. In addition, the clinical scores of group B were higher than those of group A, with scores above 30 in the typical period of clinical symptoms, and the scores of groups A and C were between 20 and 30. Moreover, the piglets in group A experienced clinical symptoms of SVA exhibited a sequential evidence.

**Figure 3:**
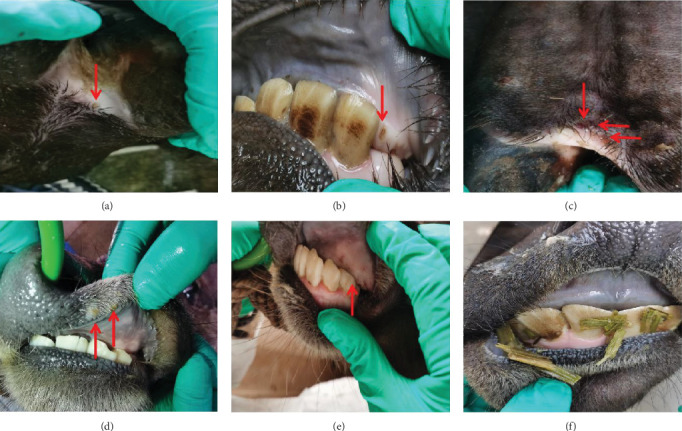
Lesions of the buffaloes. Small blisters appeared behind the coronal band of the hoof (A) and the upper alveolar ridge (B) of the number 26 buffalo, the hoof toe (C) of the number 27 buffalo, the upper lip (D) and the upper gum (E) of the number 28 buffalo. There were no blisters appeared on the buffalo of control group (F).

After infection, three buffaloes (number 23, 25, and 26) in group A and three buffaloes (number 24, 27, and 28) in group B sticked out their tongues and slapped lips and noses. Among them, the buffalo number 26 in group A blistered on the back of the coronal band of the hoof (Figure 3A), the upper alveolar ridge (Figure 3B). Blisters of different sizes appeared on the toes of the number 27 buffalo at 9 dpi (Figure 3C). The number 28 buffalo in group A first had five contiguous blisters on its upper lip at 3 dpi (Figure 3D). Two blisters on the upper gum of the number 28 buffalo were observed at 6 dpi (Figure 3E). The mental health, appetite, nose, lips, and hoof nails of the buffaloes were carefully observed and scored daily during the infection.

**Figure 4:**
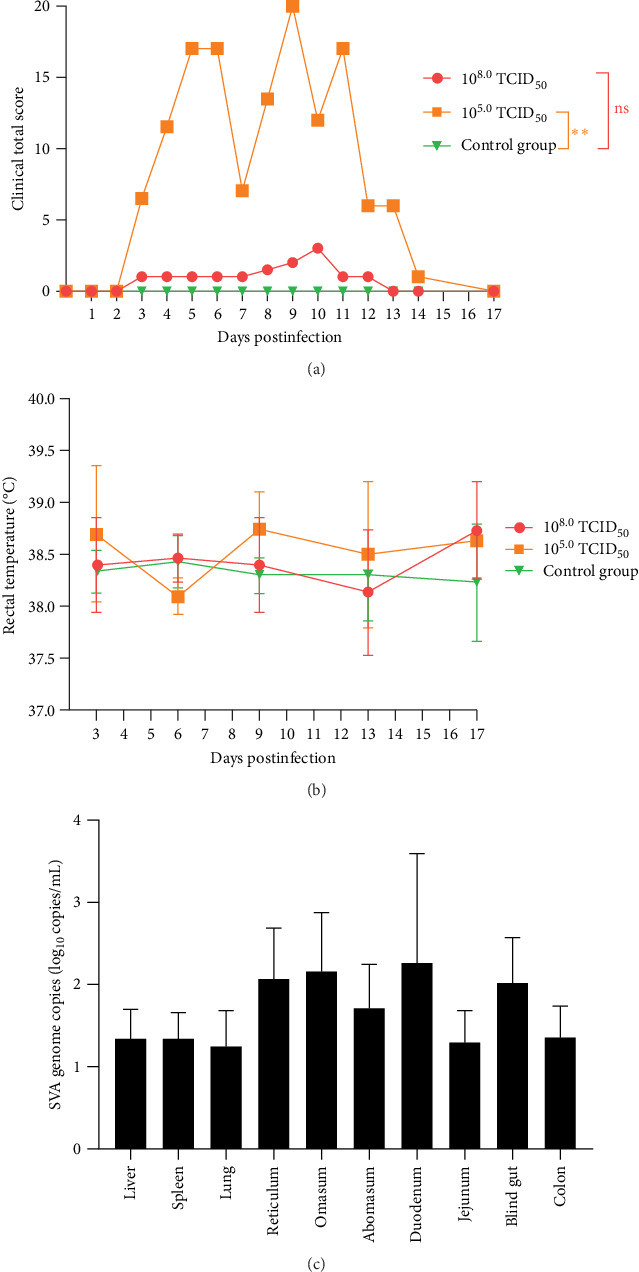
The data statistics of the buffaloes. From 4 to 11 dpi, the clinical score of the buffaloes from group B (10^5.0^ TCID_50_/mL) was more than 9, and on the 9 dpi, it was more than 18. It was also higher than that of group A (10^8.0^ TCID_50_/mL) from 3 to 14 dpi. But only the little lesions were observed, which the maximum clinical score was no more than 5 (A). However, the rectal temperature of the buffaloes in group A was slightly higher than in group B and the negative control group (B). The SVA viral loading of SVA in the gastrointestinal tract was higher than that of others, such as the liver, spleen, and lung (C).

The scores of group A were lower than those of the other groups, reaching their highest values at 10 dpi. And those of group B gradually increased from 2 dpi to the highest value at 9 dpi, and the scores ranged from 9 to 18 during the whole observation period. A comparison and analysis of the total score for each day showed that the scores for the whole experiment were greater at 5, 6 and 8–11 dpi (Figure 4A).

### 3.2. Rectal Temperature Monitor

After the piglets were infected with different SVA concentrations, the rectal temperature was measured every 2 days. No significant difference was found in the average rectal temperature (Figure 2B).

The buffaloes were infected with different virus titers of SVA, and the rectal temperature was measured at 3, 6, 9, 14, and 17 dpi. The average rectal temperature of the buffaloes in the three groups was also not significantly different (Figure 4B).

### 3.3. Changes in the Daily Body Weight of the Piglets

The body weights of all the piglets were recorded separately every 2 days from 2 to 12 dpi. The results showed that the piglets in group A had negative growth at 4 dpi. Compared with those in the control group, the body weight growth rates of the piglets in groups B and C were lower. At 8 dpi, the body growth rates of groups A and B were lower than that of the control group. At 10 and 12 dpi, the weight gain of the piglets in groups A–C was lower than that of the piglets in the control group (Figure 2C).

### 3.4. Viral Loading of Oral, Nasal, and Fecal Swabs

**Figure 5:**
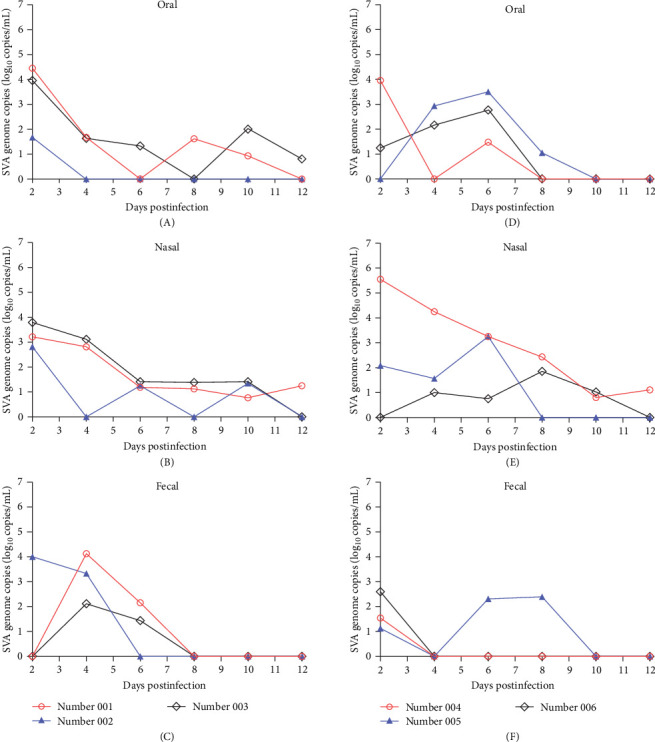
The genome copies of SVA in piglets' oral, nasal, and fecal swabs. (A–C) represent the SVA genome copies of oral, nasal, and fecal swab of piglets from group A. (D–F) represent the SVA genome copies of oral, nasal, and fecal swab of piglets from group B.

After the piglets were artificially infected with different dosages of SVA, the oral, nasal, and fecal swabs of each experimental piglet were collected and detected viral load every 2 days. The SVA gene could be detected in oral swabs until 12 dpi (Figure 5A). Nasal swabs (Figure 5B) of number 001–003 piglets from group A and oral swabs of number 003 piglet could not be detected SVA gene at 4 dpi. In contrast, the fecal swabs of three piglets could be detected SVA gene only at 6 dpi (Figure 5C). Among the piglets in group B (number 004–006), the SVA gene could be detected from the oral swabs of number 004 and number 006 at 8 dpi, but the oral swabs of number 005 could be detected SVA gene up to 10 dpi (Figure 5D). While nasal swabs from piglet number 005 could only be detected SVA gene until to 8 dpi, but the nasal swabs from piglets number 004 and 006 were still detected SVA gene at the end of the experiment (Figure 5E). The SVA gene from fecal swabs could only be detected up to 10 dpi for number 005 and 4 dpi for number 004 and 006 (Figure 5F). However, it was not detected in the oral swabs, nasal swabs, and fecal swabs of the piglets in the group C (10^3.0^ TCID_50_/mL, number 007–009) and control group D (DMEM, number 010 and 011).

**Figure 6:**
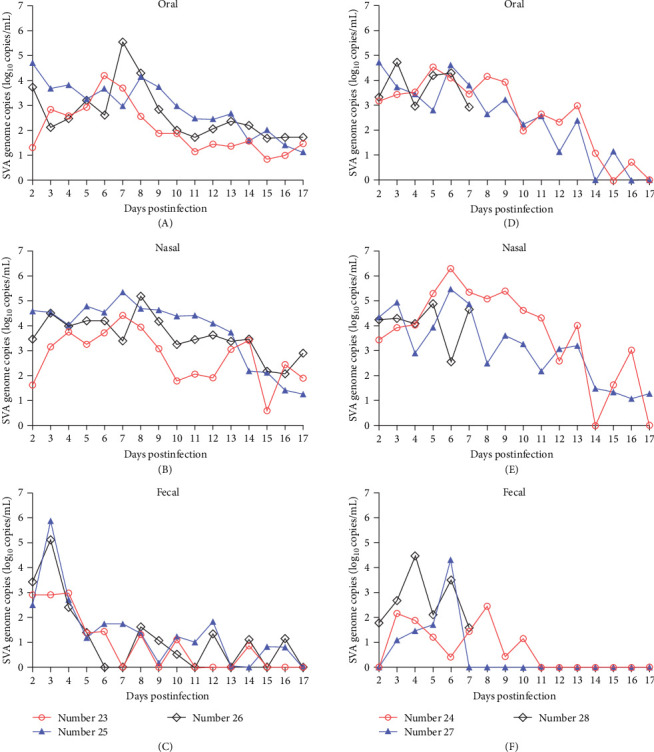
Statistics and comparison of SVA viral loading collected from the infected buffaloes. The SVA viral loading of nasal and oral swabs could be monitored more than 13 dpi (A), (B), (D) and (E), but the SVA viral loading of fecal swabs was up to peak at 3 dpi and then it was to be detected irregularly after 5 dpi (C) and (F).

For the infected buffaloes, the oral, nasal, and fecal swabs were collected every day after 2 dpi. In Figure 6, SVA genes were detected in every buffalo in group A (number 23, 25, and 26) daily. The oral and nasal viral loads peaked at 7 dpi (Figure 6A, B), while more fecal viral loads were detected only in the previous week, and rarely thereafter (Figure 6C). For the infected buffaloes in group B (number 24, 27, and 28), the SVA gene was detected in oral swabs (Figure 6D) and nasal swabs (Figure 6E) every day, and it peaked at 6 dpi. Similarly, viral loads in the feces followed the same pattern as in group A (Figure 6F). The number 28 buffalo was examined by IHC during obvious clinical symptoms, so there were no other data after 7 dpi. Normally, the SVA gene could also not be detected in the oral swabs, nasal swabs, and fecal swabs of the buffaloes in the control group C.

The number 28 buffalo exhibited typical clinical symptoms at 7 dpi. Subsequently, anatomical examination was done and apparent histopathological changes were observed. The SVA viral distribution in the heart, liver, spleen, lung, kidney, rumen, reticulum, valve stomach, abomasum, duodenum, jejunum, ileum, blind gut, colon, and rectum were detected. The results showed that the SVA gene could be detected in the liver, spleen, lung, jejunum, colon, and more in the reticulum, omasum, abomasum, duodenum, and blind gut (Figure 4C).

### 3.5. IHC Observation of Organ Pathology

IHC was used to observe SVA antigen expression in the collected lung tissue, chin blister lesion tissue, and nasolabial mirror tissue of the piglets. The results showed that SVA antigen could be detected in the lungs (Figure S1A, B), lower lip (Figure S1C, D), and rhinolabial scope lesion tissue (Figure S1E, F). The positive area intensity scores showed that the scores for chin blister lesions and nasolabial mirror tissue were greater than 80 (Table 2). Among these tissues, chin blister lesion tissue was of the highest score.

For the number 28 buffalo with typical clinical symptoms, the upper lip blister tissue was also analyzed via IHC, and the expression of the SVA antigen was also observed (Figure S1G, H). The positive area intensity score (*H*-score) was 94.184 (Table 2).

### 3.6. Virus Isolation

**Figure 7:**
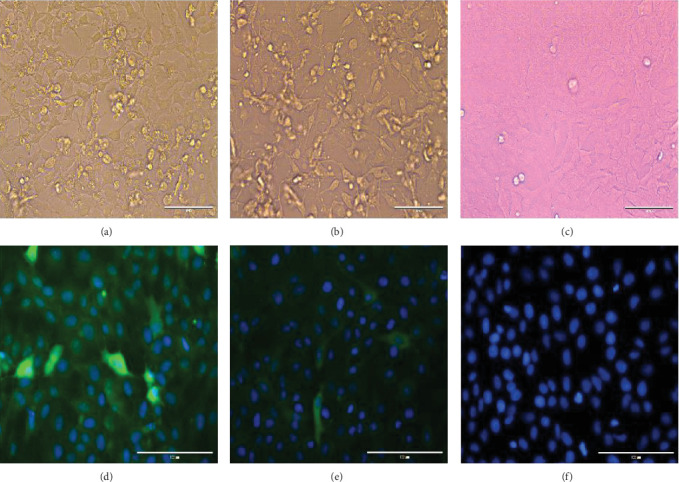
The ST cell lines infected with SVA and IFA detection. The SVA could be isolated with ST cell lines, which from the oral and nasal swabs filtrates of the piglets (A, 400 ×) and the buffaloes (B, 400 ×), followed by normal ST cell lines as MOCK (C, 400 ×). Besides, the SVA VP3 protein could be detected with IFA assay in ST cells, which infected the oral and nasal swabs filtrates of (D, 400 ×) and the buffaloes (E, 400 ×), respectively. (F) There were no specific fluorescence was observed in mock-infected cells.

The positive oral swabs from the infected piglets and buffaloes were soaked in PBS at 4°C for 1 h. The samples were filtered and transferred to ST monolayer cell lines for viral isolation. CPE was observed daily. The CPE were observed in which oral swabs from the infected piglets at 2 dpi (Figure 7A) and the buffaloes (Figure 7B). Moreover, the negative ST cells were normal (Figure 7C). The derived SVA of oral swabs from the infected piglets (Figure 7D) and the buffaloes (Figure 7E) were characterized by detection of expression of SVA VP3 markers by immunofluorescence staining.

## 4. Discussion

SVA is the only virus of the *Senecavirus* genus in the *Picornaviridae* family [19]. Natural hosts include wild boars and domestic pigs [20–24]. The feeding equipment and transportation tools used, houseflies and mice are vectors for the spread of SVA [10, 22]. To date, there were no reports about other SVA hosts. In 2018, a buffalo-origin SVA strain, SVA/GD/China/2018, was first isolated in our laboratory and its susceptibility and replication capability toward bovine-origin cell lines, such as MDBK, and swine-origin cell lines, including PK-15 and ST cell lines, were studied in vitro [14]. The results indicated that swine-origin cell lines were more susceptible and more likely to replicate than bovine-origin cell lines. To determine the difference in pathogenicity of SVA/GD/China/2018 strain in vivo, piglets and buffaloes were selected as the objects of this study.

In order to evaluate the pathogenicity of the SVA/GD/China/2018 strain on piglets and buffaloes, three different viral doses of the SVA/GD/China/2018 strain were used for artificial infection experiments. Artificial inoculation in the piglets with three viral doses of the SVA/GD/China/2018 strain, suggesting that the piglets could present typical clinical symptoms of SVA at 4 dpi with blisters in the oral cavity, nasal mirror, coronet, surrounding area, some hoof nail shedding, putrefaction, and necrosis, and slower weight growth rates and generally higher rectal temperature than those of the control group. These clinical symptoms were similar to those of the pigs infected artificially or naturally by swine-origin SVA. The clinical score results were similar to those of published studies, and the highest score was between 4 and 8 dpi [21]. In this study, the group with the highest clinical score in the SVA infection group was the medium-viral-dose group, followed by the high-viral-dose group and the low-viral-dose group, suggesting that the clinical symptoms of piglets infected with SVA were related to the virus dose. The group with the lowest viral dose (10^3^ TCID_50_/mL) did not cause significant clinical manifestations. Therefore, the results of the present study indicated that an appropriate viral dose can result in persistent typical clinical symptoms. The oral, nasal, and fecal swabs of the experimental piglets were collected every 2 days to detect SVA gene copies. There were no obvious regular changes. Generally, virus shedding was observed in piglets in the high-viral-dose group and the medium-viral-dose group at nearly 7 dpi, indicating that the piglets inoculated artificially did not exhibit regular virus shedding.

The SVA/GD/China/2018 strain used in this study was first isolated from the buffalo. According to Koch's postulates, the pathogenicity on this strain was conducted on buffaloes. The clinical characteristics during the whole study were similar to those of piglets infected with SVA/GD/China/2018. The blisters were observed on the lips, mouths and hoof nails of some buffaloes. Significant difference in rectal temperature or similar infection dynamics was not monitored between the virus-infected groups and the control group [25]. While the buffaloes were infected with the SVA/GD/China/2018 strain, there was greater virus shedding in the oral and nasal swabs in the two groups than in the fecal swabs, and the number of SVA gene copies in the nasal and oral swabs was greater than that in the fecal swabs, which indicated that the nasal and oral pathways were the main virus shedding pathways for the buffaloes. The SVA antigen expression in the tissues of the number 28 buffalo indicated that the gastrointestinal tract had a higher viral load. It is worth noting that, for one thing, the difference in clinical symptoms between the buffaloes and the piglets infected with the SVA/GD/China/2018 strain was reflected in the absence of hoof nail shedding; for another thing, when compared with the primary buffaloes, typical clinical symptoms of SVA, such as blisters, have been able to develop on the buffaloes infected with the SVA/GD/China/2018 strain, indicating that the SVA/GD/China/2018 strain was pathogenic. Moreover, the pathogenicity of the strain for buffaloes was slighter than that for piglets. The occurrence of typical clinical symptoms is also related to the SVA dose [26, 27].

The SVA/GD/China/2018 strain is the first cross-host propagation pathogenic strain in which typical clinical symptoms are observed in at least half of buffaloes. The pathogenicity of SVA in cattle may increase. The key amino acid change could adapt the new strains to new hosts for replication and proliferation [28], which helps to increase its infectiousness [29–31]. The SVA strains with high pathogenicity to buffaloes even other new hosts would emerge after long-term evolution and selection. The first porcine-origin SVA strain, SVV-001 which isolated in 2002 can infect pigs but can not initially lead to obvious pathogenicity except for a slight mental state [1]. However, SVA strains isolated from 2002 to 2008 could caused typical clinical symptoms in pigs. More seriously, since 2015, many SVA outbreaks have occurred on pig farms in China and the United States [4, 32–35].

The pathogenicity of SVA in pigs become increasingly serious with increasing variation in certain genes. Over 10 recombinant porcine SVA strains have been found by five recombinant patterns, including the SVA/GD/China/2018 strain in this study, which is also a recombinant SVA strain between the American and Chinese strains. The virulence of each SVA strain is different [26, 36, 37]. In this study, the SVA/GD/China/2018 strain did not cause the death of buffaloes from 0 to 17 dpi. The histopathological observation and viral loading detection within the organization were finished for only one buffalo with severe clinical symptoms, while other buffaloes were used to observe long-term clinical symptoms. After 2 months of observation, no buffalo died or exhibited clinical symptoms similar to those of SVA, which indicated that the infection kinetics of this strain in buffaloes were similar to those of porcine-derived SVA strains in pigs. However, one recent study showed that a porcine-derived SVA (NADC4, GenBank number MZ733977) is not susceptible to colostrum-deprived Holstein calves [38]. The main reason could be explained by the origin of SVA and the adaptation time of the different hosts, the initial samples of the SVA/GD/China/2018 strain were from buffaloes with clinical characteristics.

The results here confirmed that buffalo may be another host of SVA. Nevertheless, additional studies are needed to confirm whether porcine-derived SVA can cause typical clinical symptoms in buffaloes, beef cattle or cows, which should be considered and monitored for cross-host spread. Also, it is important to strengthen the identification of diagnostic criteria for distinguishing between FMDV and SVA because of the similarity of vesicular lesions [39].

## 5. Conclusion

In conclusion, the study systematically evaluate the pathogenicity of the buffalo-origin SVA strain, SVA/GD/China/2018, to the buffaloes and piglets in animal experiment conditions, suggesting the cross-species transmission potential of SVA. In the future, it is necessary to strengthen the surveillance of SVA in cattle herds.

## Figures and Tables

**Table 1:** The clinical scoring reference.

Observation subject	Status	Scores
Mental status	Normal	0
	Depressed	0.5
	Anorexic	2
	Dying	3

Breathing problems	Normal	0
	Mild dyspnea	0.5
	Moderate dyspnea	1
	Severe dyspnea	2

Vascular damage	Normal	0
	Congestion or scab	1
	Red and swollen blister	2
	Ulcerative lesion or suppuration	3
	Hoof nail detachment	4

**Table 2:** IHC score of the infected tissue samples.

Species	Tissue	Positive area (%)	Mean density	Areal density	*H*-score
Piglets	A–B: lung	33.3152	0.0948	0.031585	84.6661
	C–D: lower lip	30.0377	0.0667	0.020049	104.0242
	E–F: rhinolabial scope lesion	19.8384	0.0855	0.016963	80.4032

Buffaloes	Number 28 upper lip lesion	47.194	0.074	0.034944	94.184

## Data Availability

The SVA/GD/China/2018 genome was deposited in the National Center for Biotechnology Information database, https://www.ncbi.nlm.nih.gov/nucleotide/, and the accession number is MN615881.

## Supporting Information

Supporting Information Figure S1. Histopathological examination of infected tissue samples lung, lower lip, rhinolabial scope lesion tissue of the infected piglets, and the upper lip of the infected number 28 buffalo. The SVA antigen expressed in the lung (A [6×] and B [400×]), lower lip (C [1.3×] and D [400×]), and rhinolabial scope tissue (E [2.6×] and F [400×]) of the infected piglets. The SVA antigen expressed on the upper lip (A [6×] and B [400×]) of the infected buffalo.
