# Supplementary material for: Pathogenicity of the First Buffalo-origin Senecavirus A in Conventional Piglets and Buffaloes
Source: Transbound Emerg Dis. 2025 Sep 2;2025:6222217. doi: 10.1155/tbed/6222217 (PMC12419930; doi:10.1155/tbed/6222217)
Supplement: Supporting Information — Figure S1. Histopathological examination of infected tissue samples lung, lower lip, rhinolabial scope lesion tissue of the infected piglets, and the upper lip of the infected number 28 buffalo. The SVA antigen expressed in the lung (A [6×] and B [400×]), lower lip (C [1.3×] and D [400×]), and rhinolabial scope tissue (E [2.6×] and F [400×]) of the infected piglets. The SVA antigen expressed on the upper lip (A [6×] and B [400×]) of the infected buffalo. [file 6222217.f1.pdf]

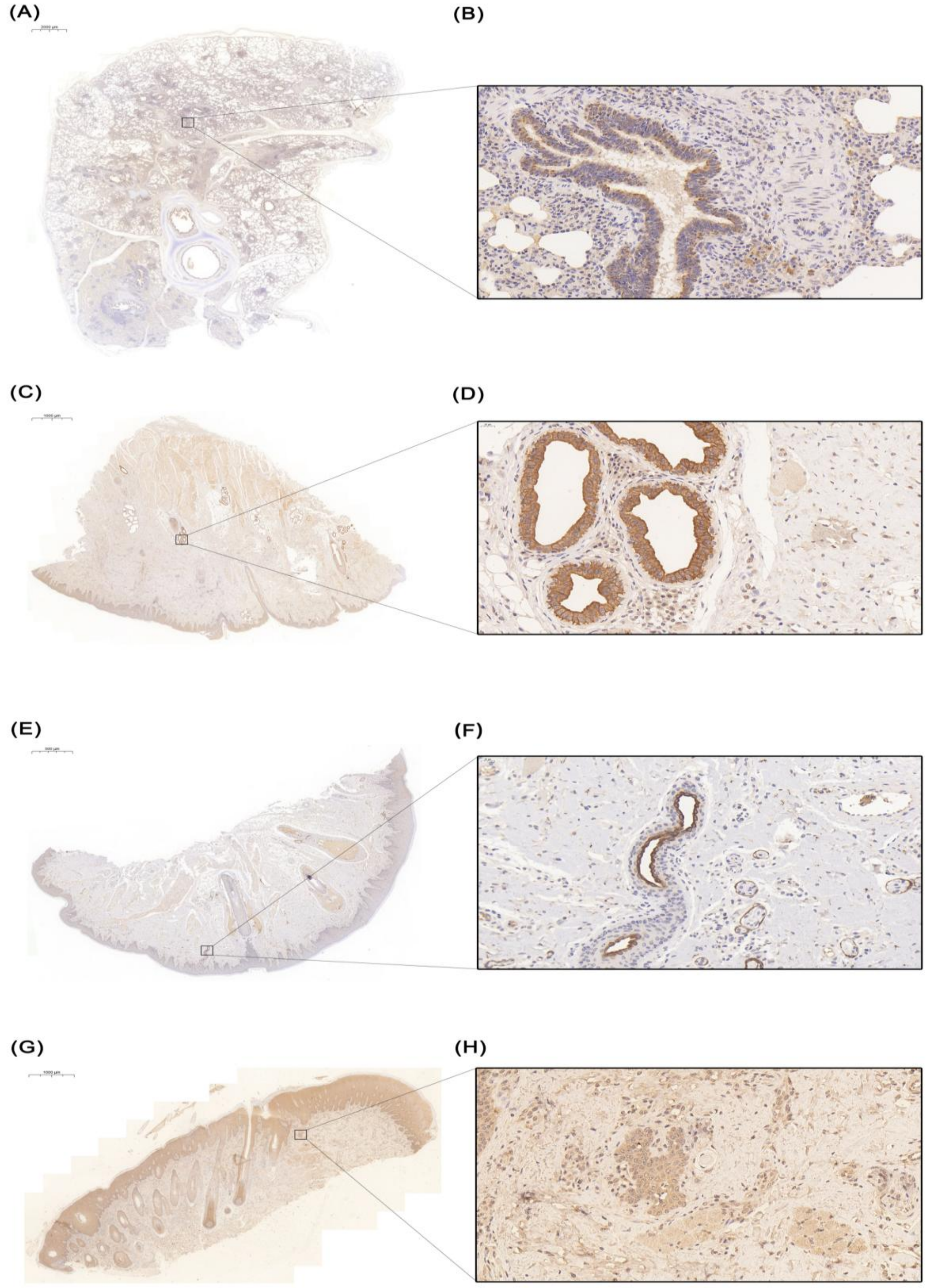

Figure S1. Histopathological examination of infected tissue samples lung, lower lip, rhino-labial scope lesion tissue of the infected piglets and the upper lip of the infected No.28 buffalo. The SVA antigen expressed in the lung (A, 6× and B, 400×), lower lip (C, 1.3× and D, 400×), and rhino-labial scope tissue (E, 2.6× and F, 400×) of the infected piglets. The SVA antigen expressed on the upper lip (A, 6× and B, 400×) of the infected buffalo.
